# Assessment of Mixed-Reality Technology Use in Remote Online Anatomy Education

**DOI:** 10.1001/jamanetworkopen.2020.16271

**Published:** 2020-09-17

**Authors:** Susanne Wish-Baratz, Andrew R. Crofton, Jorge Gutierrez, Erin Henninger, Mark A. Griswold

**Affiliations:** 1 School of Medicine, Case Western Reserve University, Cleveland, Ohio; 2 Interactive Commons, Case Western Reserve University, Cleveland, Ohio

## Abstract

This survey study uses survey responses from first-year medical students to evaluate the initial experience of an anatomy lesson delivered remotely using teleconferencing and mixed-reality technology.

## Introduction

The coronavirus disease 2019 (COVID-19) pandemic has presented challenges for education worldwide, especially in medical schools that rely on cadaver-based dissection for anatomy. The advent of commercial mixed-reality (MR) technology, such as the HoloLens (Microsoft Corporation), offers new possibilities for anatomy education.^1^ At Case Western Reserve University (CWRU), the state of Ohio’s shelter in place order meant that students did not return from spring break in 2020, requiring an urgent modification to the anatomy curriculum, which has featured MR technology since 2018.^2^ We report our initial experience using MR to teach anatomy remotely to students located throughout North America.

## Methods

This survey study used a modification of the HoloAnatomy Software Suite (CWRU)^2^ that allowed headsets to communicate across different Wi-Fi networks and a system to allow instructors and students to digitally point at an object to ask and answer questions. The CWRU institutional review board classified this study as exempt because the survey responses were completely anonymized. Informed consent was implied by completion of the survey. The study followed the American Association for Public Opinion Research (AAPOR) reporting guideline.

On March 11, 2020, CWRU began shipping MR headsets to all 185 students in the CWRU School of Medicine first-year class. To minimize the potential for COVID-19 transmission, devices were cleaned with 70% isopropyl alcohol and exposure to high intensity UV-C light for 1 minute (Cleanbox) and individually shipped overnight. The 185 students who participated in the study were divided into 4 groups. Each group attended an equivalent 50-minute anatomy lesson focused on the bronchi, lungs, vasculature, and lymphatics. The lessons were held on 2 separate days (March 24 and 26, 2020). Real-time audio and chat were transmitted using the Zoom teleconferencing application (Video Communications, Inc). Class content lasted 35 minutes, with the remaining time set aside for potential technical issues. After each session, students were sent a survey to assess their experience and the technology’s performance. Statistical analysis of the survey responses was performed using Excel (Microsoft Corporation).

## Results

All 185 students successfully completed the anatomy sessions. As shown in the Table, 177 students (96%) responded to the postinstruction survey. No demographics were recorded from respondents, but the class comprised 95 women and 90 men aged 21 through 34 years. Following AAPOR guidelines, all returned surveys were considered complete, and we classified nonresponders as participants of unknown eligibility. Only 28 students (16%) reported experiencing technical issues that they had not previously experienced in the in-person class, whereas 143 (81%) reported that the remote anatomy sessions were equivalent to or better than the in-person class. When given a choice, 102 students (58%) preferred remote delivery to in-person classes, and 148 (84%) reported believing that students can effectively learn human anatomy using this remote MR application. A total of 143 respondents (81%) reported seeing advantages of remote sessions compared with in-person sessions. Analysis of the 131 qualitative responses about remote sessions showed that the most common advantages reported by students were the ability to study on their own time (67 [51%]) and having more physical space to move around the anatomical models (32 [24%]). The most common disadvantage mentioned was difficulty interacting with the teacher and other students to ask questions (32 [24%]).

**Table.** Responses to a Survey by 177 Students About a Remote Anatomy Lesson Using HoloAnatomy^a^

Survey item	Responses, No. (%)
How would you rate your experience with the remote HoloAnatomy session compared with the in-person HoloAnatomy sessions?	
Much better	17 (10)
Better	34 (19)
Equivalent	92 (52)
Worse	30 (17)
Much worse	4 (2)
After experiencing a remote mixed-reality session, do you think that students can effectively learn anatomy via HoloAnatomy remotely?	
Yes	148 (84)
No	4 (2)
Unsure	25 (14)
Which form of HoloAnatomy session do you prefer?	
Remote	102 (58)
In-person	75 (42)
Did you have any technological issues with the remote HoloAnatomy session?	
Yes	41 (23)
No	136 (77)
Did you experience any significant issues with the remote HoloAnatomy session that you have not experienced with in-person HoloAnatomy sessions?	
Yes	28 (16)
No	149 (84)
Do you see any advantages with remote HoloAnatomy sessions vs in-person HoloAnatomy sessions?	
Yes	143 (81)
No	34 (19)

^a^ HoloAnatomy Software Suite (Case Western Reserve University).

## Discussion

Previous studies have found that students achieve the same level of acquired knowledge in approximately half the time using MR in both medical^1,2,3^ and nonmedical situations.^4^ To our knowledge, this study was the first to show positive learning experiences for an entire class of medical students using MR remotely. The primary limitation of this study is that it was conducted at only a single institution and with students who had prior MR experience.

Outside the current pandemic, these results also address an everyday situation affecting students worldwide owing to the cost of maintaining cadaver laboratories, the lack of qualified faculty, or other societal reasons. The minimum required infrastructure to run applications, such as HoloAnatomy, is an MR headset and basic Wi-Fi connectivity. Similar to most computer technology, MR headsets are likely to become more affordable over time. Thus, MR has the potential to address cost and access issues to enable high-quality medical education around the world.
